# Assessing the Nature of Chiral-Induced Spin Selectivity by Magnetic Resonance

**DOI:** 10.1021/acs.jpclett.1c01447

**Published:** 2021-07-06

**Authors:** A. Chiesa, M. Chizzini, E. Garlatti, E. Salvadori, F. Tacchino, P. Santini, I. Tavernelli, R. Bittl, M. Chiesa, R. Sessoli, S. Carretta

**Affiliations:** † Dipartimento di Scienze Matematiche, Fisiche e Informatiche, Università di Parma, I-43124 Parma, Italy; ‡ UdR Parma, INSTM, I-43124 Parma, Italy; ¶ Dipartimento di Chimica & NIS Centre, Università di Torino, Via P. Giuria 7, I-10125 Torino, Italy; § IBM Quantum, IBM Research—Zurich, Säumerstrasse 4, 8803 Rüschlikon, Switzerland; ∥ Freie Universität Berlin, Fachbereich Physik, Berlin Joint EPR Lab, Arnimallee 14, D-14195 Berlin, Germany; ⊥ Dipartimento di Chimica “Ugo Schiff” & INSTM, Università Degli Studi di Firenze, I-50019 Sesto Fiorentino, Italy

## Abstract

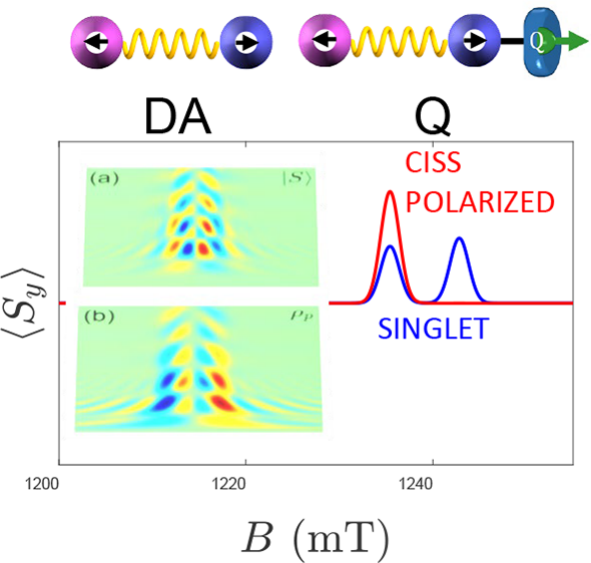

Understanding chiral-induced spin selectivity (CISS), resulting from charge transport through helical systems, has recently inspired many experimental and theoretical efforts but is still the object of intense debate. In order to assess the nature of CISS, we propose to focus on electron-transfer processes occurring at the single-molecule level. We design simple magnetic resonance experiments, exploiting a qubit as a highly sensitive and coherent magnetic sensor, to provide clear signatures of the acceptor polarization. Moreover, we show that information could even be obtained from time-resolved electron paramagnetic resonance experiments on a randomly oriented solution of molecules. The proposed experiments will unveil the role of chiral linkers in electron transfer and could also be exploited for quantum computing applications.

Charge displacement through chiral systems has been suggested as a resource for spintronics devices and as the driving force of many biological reactions.^1−3^ This has led to huge research efforts, mainly focused on the detection of a spin-polarized current^4^ filtered by chiral molecules, a phenomenon known as chiral-induced spin selectivity (CISS). Transport experiments were done on self-assembled monolayers of chiral (χ) molecules or on individual molecules addressed by atomic force microscopy.^5−8^ Additional studies revealed polarization also in very different contexts, in which no steady-state current flows through chiral molecules (see the Supporting Information and refs (2 and 9−14)). In parallel, various theoretical models have been put forward,^15−31^ but a comprehensive, even qualitative, description of this widespread phenomenon is lacking.^8^

To shed light on the origin of CISS and build a satisfactory theoretical model, we still miss some detailed information on the spin wave function, after an electron has crossed a chiral bridge. This can be achieved by simplifying the experimental setup and focusing on qualitative features, emerging directly from chiral molecules. In particular, electron-transfer (ET) processes through a chiral bridge linking a donor and an acceptor (D-χ-A in the following, see bottom inset of Figure 1) may serve as the ideal platform to understand this phenomenon, in which all other complex elements (such as leads, interfaces, and substrates) have been removed. Recently, a minimal model of ET in chiral environments was proposed, in which the bridge was included via an effective spin–orbit interaction. This leads to no local spin polarization on D/A starting from the singlet state precursor obtained by photoexcitation (PE).^32^ In contrast, experiments on photosystem-I^1,33^ have demonstrated a spin polarization occurring also in ET.

**Figure 1:**
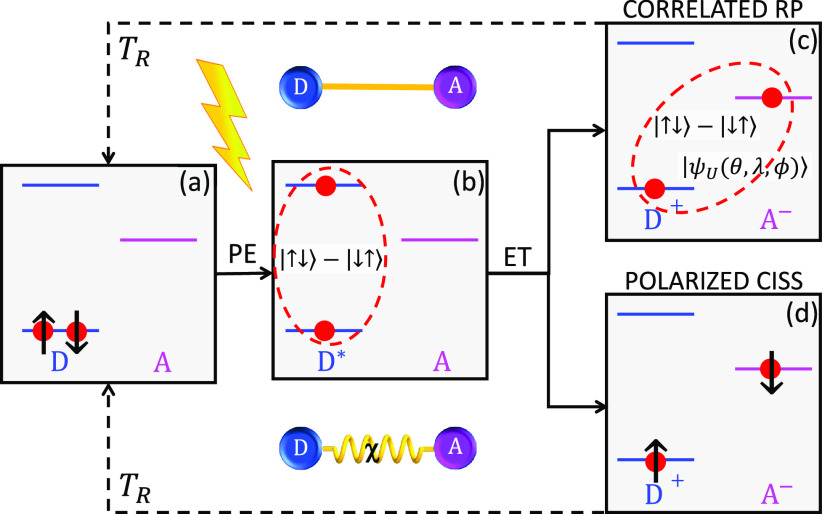
Scheme of the electron-transfer mechanism: (a) singlet initial state on the donor D (with two electrons both in the ground orbital). Photoexcitation (PE) brings it to the D*A singlet state in which one electron is excited (b), but the pair is still in an entangled state (dashed circle). After electron transfer (ET) of the excited electron to the acceptor (A), the final state is still either a correlated radical pair (RP, c) or a polarized state after transfer through a chiral bridge (d). Recombination to the initial singlet (or to the triplet) state occurs on a time scale *T*_R_ (dashed arrows). Top (bottom) inset: Scheme of the DA radical pair, linked by a linear (chiral) bridge.

We propose here simple experiments to unambiguously distinguish the two situations, thus finally elucidating the nature of CISS, by answering the question: is the electron spin polarized after ET through the chiral bridge? The experiments are based on using a highly coherent qubit (Q), coupled to the acceptor in a D-χ-A-Q setup, as a local probe of this polarization transfer and time-resolved electron paramagnetic resonance (TR-EPR) as the experimental tool. By acting as an external and local sensor, the qubit gives direct access to the acceptor polarization, without influencing the ET process. This provides a unique means to assess the nature of CISS at the single-molecule level, much more directly compared to previous setups,^1^ where many additional ingredients could somehow obscure the role of the χ unit. The second experiment we propose probes the qubit state after polarization has been coherently transferred from A to Q by an appropriate pulse sequence. We show that both approaches yield unambiguous fingerprints of the polarization of the acceptor if implemented on an oriented solution of D-χ-A-Q molecules. Moreover, we demonstrate by numerical simulations that features of CISS can also be observed in TR-EPR spectra of a much simpler experimental setup consisting of a randomly oriented ensemble of D-χ-A molecules. The know-how reached by performing the proposed experiments will be the starting point to develop a sound theoretical model of CISS.

*D-χ-A System*. We consider the following experimental scenario, schematically shown in Figure 1: the donor, initially in a doubly occupied ground state (panel a) is photoexcited to the D*-χ-A singlet state (b). Then, ET yields the charge-separated state D^+^-χ-A^–^. In the case of a linear bridge linking donor and acceptor (top inset), this is still a two-electron singlet state (Figure 1c). Our aim is to compare this situation, typical of a spin-correlated radical pair (RP),^34−40^ with that in the presence of a chiral bridge. In particular, in the experiments proposed below we consider the following charge-separated states:

(1) A singlet state, , typical of ET through a linear achiral bridge.

(2) A polarized state, represented by the density matrix ρ_*p*_ =  (with −1 ≤ *p* ≤ 1 and ≠0). Here *p* = −2Tr[ρ_*p*_*S*_*zA*_] is the final polarization of the acceptor. This state (represented in Figure 1d for *p* = 1) could result from spin selective ET through a chiral bridge, as found in measurements on photosystem-I^33^ and in other experiments,^9−11,13,41^ where charge polarization was induced by application of an electric field, thus making these situations somewhat similar to ET.

(3) A non-Boltzmann but nonpolarized (correlated) state |ψ_*U*_⟩ (Figure 1c) resulting from a coherent rotation of the transferred electron belonging to , as proposed in ref (32). The most general form of this state is given by |ψ_*U*_⟩ = . One can easily check that |ψ_*U*_⟩ does not give any local (one-)polarization, i.e., ⟨ψ_*U*_ | *S*_*zD*_ | ψ_*U*_⟩ = ⟨ψ_*U*_ | *S*_*zA*_|ψ_*U*_⟩ = 0.

The three possible ET outputs are clearly discriminated by the experiments proposed below.

*Detecting Polarization Using a Qubit Sensor*. To detect spin imbalance in the D^+^-χ-A^–^ unit, we propose the use of a qubit, i.e., a paramagnetic *S* = 1/2 center showing long coherence. Molecular spin qubits are very promising sensors^43^ thanks to the very long coherence times they can reach if properly chemically engineered.^44−58^ In addition, the capability to link these qubits to other units make them ideal candidates for the proposed architecture. As a model example, we consider a qubit based on the VO^2+^ unit, which has already demonstrated remarkable coherence even at room temperature.^50^ Interestingly, a setup consisting of a chain of three radicals was studied in refs (59−64).

After ET, the whole D-χ-A-Q system is described by the following spin Hamiltonian:
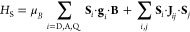
1where the first term models the interaction of each of the three spins with an external magnetic field, while the second describes the magnetic dipole–dipole interaction between D–A and A–Q. We assume the point-dipole approximation, leading to **J**_*ij*_ = [**g**_*i*_ ·**g**_*j*_ – 3(**g**_*i*_ · **r**_*ij*_)(**g**_*j*_ · **r**_*ij*_)]μ_*B*_^2^/*r*_*ij*_^3^, and in the following we consider for simplicity a linear 3-spin chain (see Figure 2a), with *r*_DA_ = 25 Å and isotropic *g*_D,A_. As demonstrated by simulations reported in the Supporting Information, possible additional isotropic exchange contributions to **J**_AQ_ do not alter our conclusions, provided that *J*_AQ_^*x*,*y*^ is sufficiently smaller than | *g*_A_ – *g*_Q_^*z*^|μ_*B*_*B* to make the initial state of the qubit factorized from that of the acceptor. We note, in turn, that a stronger A–Q coupling could be compensated either by increasing *B* or by choosing a different qubit, based for instance on Cu^2+^,^52^ yielding a larger | *g*_A_ – *g*_Q_^*z*^|. In this regime, Q acts as a coherent quantum sensor which does not perturb the RP but only detects local spin polarization. At the same time, *J*_AQ_^*z*^ should be larger than the line width corresponding to Q excitations. For instance, by choosing fwhm = 2.35 mT (a conservative estimate for typical transition metal ion based qubits^42,49^), these conditions are easily fulfilled with 6 Å ≲ *r*_*AQ*_ ≲ 11 Å and working in Q-band. We thus fix *r*_AQ_ = 8 Å in the following simulations.

**Figure 2:**
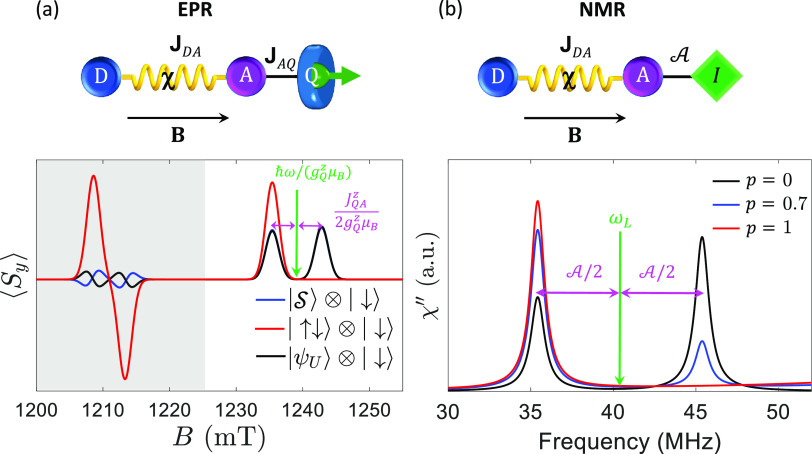
(a) Q-band TR-EPR spectrum of a D^+^-χ-A^–^-Q system (sketched on top) with the chiral axis aligned parallel to the external field (integrated from 100 to 300 ns) and for different initial states of the radical pair (with the qubit always in |↓⟩): singlet (blue line), corresponding to transfer along a linear bridge without CISS effect; fully polarized state (on both donor and acceptor, red); unpolarized state |ψ_U_⟩ as suggested in ref (32), black. The gray-shaded area represents the signal from the donor–acceptor, while at larger field the absorption peaks are due to the qubit. (b) NMR spectrum as a function of frequency, probing nuclear excitations on a nuclear spin 1/2 (e.g., a ^19^F, Larmor frequency ν_L_ ≈ 40 MHz at 1 T) coupled by hyperfine interaction to the donor. The different intensity of the two peaks for *p* = 0 is due to different matrix elements for the two transitions. Variance from the *p* = 0 behavior directly measures the acceptor polarization. Parameters: *hν* = 34 GHz, *J*_AQ_^*z*^ ≈ 200 MHz, *r*_DA_ = 25 Å, *r*_AQ_ = 8 Å,  = 10 MHz, *g*_1,2_ = *g*_e_ ∓ Δ*g*/2, with Δ*g* = 0.002, **g**_*Q*_ = (1.98, 1.98, 1.96), as typical for VO^2+^ or Ti^3+^,^42^*T*_1_ = 2 μs, *T*_2_ = 0.5 μs, and *T*_R_ = 10 μs. Inhomogeneous broadening of the parameters is included by a Gaussian broadening of the peaks with fwhm 2.35 mT. To generalize our analysis, we did not include parameters of a specific qubit, such as hyperfine interaction.

Two different experiments exploiting the qubit as a sensor of the acceptor polarization are proposed. The first consists of TR-EPR measurements recorded immediately after ET. To simulate TR-EPR spectra, we compute the time evolution of the system density matrix by integrating the Liouville equation ρ̇ = , where ρ is the system density matrix in the rotating frame and *H̃*, *R̃*, and *K̃* are superoperators associated with the system Hamiltonian (including also a continuous-wave oscillating field) and with phenomenological incoherent mechanisms. These are (i) relaxation, (ii) dephasing, and (iii) recombination (see the Supporting Information and, for example, refs (36−40 and 65)), parametrized by the characteristic times *T*_1_, *T*_2_, and *T*_R_, respectively, treated as independent for each of the three spins.^61^ We use conservative values (even at room temperature) for each of the three spins of *T*_1_ ≈ 2 μs, *T*_2_ ≈ 0.5 μs, and *T*_R_ ≈ 10 μs.^37,38,50^ The precise value of these parameters yields only a broadening of the peaks in the field-dependent spectrum and a damping in the time evolution, as shown by detailed simulations in the Supporting Information. However, as far as these characteristic times are above a few hundred nanoseconds, they practically do not affect our conclusions. The recorded signal then corresponds to ⟨*S*_*y*_(*t*, *B*)⟩ = Tr[∑_*i*_*S*_*yi*_ρ(*t*)]. In order to unambiguously unveil the nature of CISS, we consider oriented D-χ-A-Q molecules, with the static field parallel to the chiral axis, *z* (experimentally achieved for instance by poling, thanks to the large electric dipole moment typical of chiral molecules based on oligopeptides^24,66−68^).

The three states (1–3) give distinct TR-EPR signals, as shown in the time-integrated spectra of Figure 2a. In particular, we note that different (unpolarized) states, such as  or |ψ_*U*_(θ, ϕ, λ)⟩, modify the radical-pair spectrum (black vs blue curves in the gray-shaded area) but not the qubit response (right part of the spectrum), which is affected only by ⟨*S*_*zA*_⟩. The qubit absorption peak close to ∼1.24 T is split by the interaction with the acceptor. If the latter is completely polarized (e.g., in |↓⟩_A_ state, *p* = 1), a single peak appears, corresponding to the |↑↓↓⟩ → |↑↓↑⟩ transition.^77^ Conversely, an unpolarized DA state induces the additional excitation corresponding to |↑⟩_A_ with approximately the same intensity (apart from slightly different matrix elements or thermal population). Note that in the present simulation, ⟨*S*_*y*_(*t*, *B*)⟩ shows a weak time dependence (see the Supporting Information), thus making the choice of the time window of integration not crucial.

Similar information can be obtained by broadband NMR spectroscopy, as reported in Figure 2b. We consider, in this case, a nuclear spin *I* = 1/2 (such as ^19^F) coupled to A by (isotropic) hyperfine interaction . The NMR absorption signal (χ″) is shown as a function of frequency, in the range corresponding to the excitation of nucleus *I*, split by hyperfine interaction with A. Again, the probe is only sensitive to ⟨*S*_*zA*_⟩ and weakly perturbs the system, thus giving direct access to the acceptor polarization.^78^

Partial polarization leads to intermediate situations (blue curve in Figure 2b and the Supporting Information), thus making the relative intensity of the two peaks a measure of spin polarization. Remarkably, both for EPR and NMR experiments, this feature is not hampered by performing the experiment at high temperature, which only induces an overall attenuation of the signal (see simulation in the Supporting Information). Moreover, opposite polarization (arising in model 2 by changing the enantiomer) yields inversion of the intensity of the two peaks, thus providing direct proof of the occurrence of CISS, in contrast to other polarization mechanisms such as chemically induced dynamic electron polarization (CIDEP).^69^

The second experiment is based on a sequence of pulses properly designed to coherently transfer polarization from A to Q, followed by EPR measurement of the final state of the system. The sequence is reported in Figure 3a,b: it consists of a first π-pulse on A (conditioned by Q state, purple arrow), followed by a π-pulse on Q, conditioned by the state of A (green arrow). Starting with the qubit in a complete mixture,  (high-temperature limit), and a fully polarized acceptor state, this sequence completely transfers polarization to Q, leaving A in an unpolarized mixture. The final state of the system can be measured by EPR after the pulses. The resulting ⟨*S*_*y*_⟩ is reported in Figure 3c, for the case of an initially polarized acceptor state (red, blue lines), compared to an initially unpolarized one (|ψ_*U*_⟩ or , black). The latter give a very weak signal, both at low field (from the radical pair) and at high field (from the qubit). Conversely, starting from a polarized |↓_D_ ↑_A_⟩ state, the final state is also polarized on both D and Q. The peaks corresponding to excitations of Q and D are then split by **J**_AQ_ and **J**_DA_, respectively (the latter being much smaller and hence not visible in Figure 3c). Note that by changing the enantiomer the absorption spectrum changes sign, due to reversal of the initial state polarization (blue trace in Figure 3c). This provides clear proof that the net spin polarization arises from CISS. Hence, a qubit (or a nuclear spin) weakly interacting with a D-χ-A unit is the ideal probe of the spin imbalance on the DA pair and would unveil the nature of CISS. We also point out that the proposed platform is robust even at room temperature, a condition in which many χ units keep large polarization efficiency^2^ and VO^2+^ qubits maintain remarkable coherence.^50^

**Figure 3:**
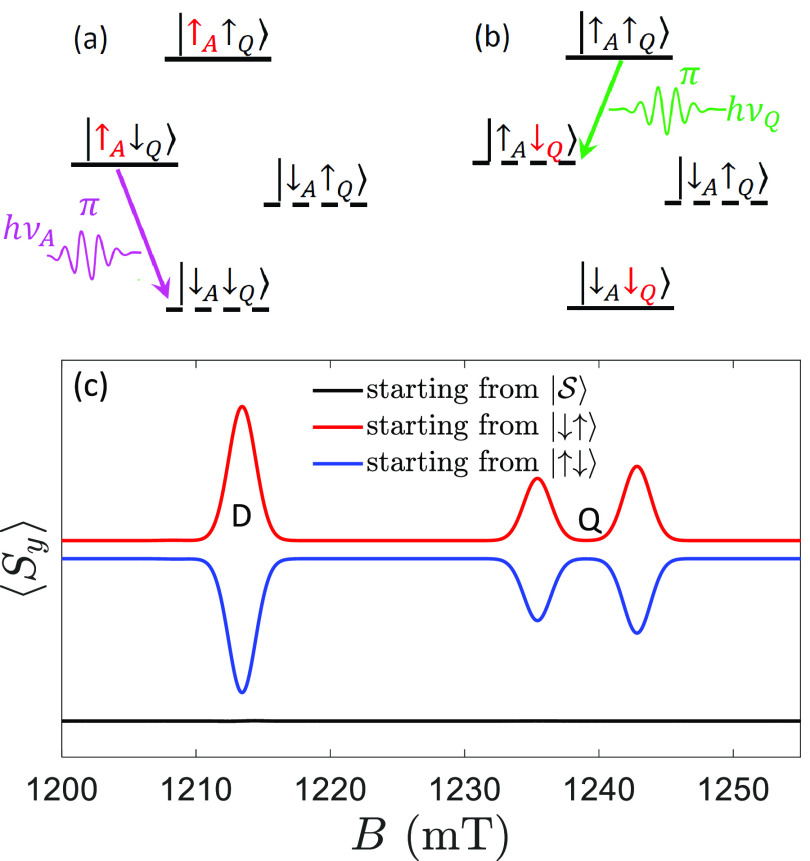
Polarization transfer to the qubit probe: (a) and (b) Pulse sequence implementing the scheme on the AQ pair (D has opposite polarization compared to A and is not affected by the pulses; that is, rotations of A are independent from the state of D). Full (dashed) lines indicate occupied (empty) states, initially with fully polarized A, finally with polarization transferred to Q (red arrow). The two pulses on A (Q) are indicated by a purple (green) arrow. (c) TR-EPR spectrum (integrated from 100 to 300 ns) after application of the polarization transfer sequence for an unpolarized state (black line) or for a spin polarized one (red or blue, depending on the polarization), as expected after CISS induced by each of the two enantiomers. Transitions involving excitations of D (Q) are represented by peaks at low (high) field.

*TR-EPR on D-χ-A in Solution*. In order to facilitate the first experimental attempts, we further simplify our setup and consider TR-EPR experiments^36−38,62,63,70^ on an isotropic solution of D-χ-A molecules. It was recently pointed out^71^ that angular average on the initial state cancels the most clear signature of CISS. However, we find that, in the presence of an anisotropic dipolar DA interaction, characteristic features of CISS are already present in the spectrum of an isotropic solution of D-χ-A molecules. In particular, the different initial states discussed in the previous sections lead to different time evolutions and hence to significantly different spectra at short times. As an example, Figure 4 shows simulated TR-EPR spectra at 9.8 GHz (X-band), along with cuts for specific time/field windows. We immediately note that at short times the  state (panel a) gives an opposite pattern of maxima and minima, compared with ρ_*p*_ (panel b). This also emerges from the short-time spectra, as a function of *B* (panel c), and from the time dependence, reported in panel d for *B* corresponding to the first pronounced peak. The different order of maxima and minima as a function of *B* can be understood by considering the form of the initial state along different directions. An illustrative diagram of the (practically factorized) eigenstates is shown in the inset of Figure 4d, with levels labeled in order of increasing energy from 1 to 4 and allowed transitions indicated by dashed lines. Note that the corresponding gaps and resonance fields are made different by **J**_DA_. An initial  state shows spherical symmetry and hence keeps the same form in any direction. The static Hamiltonian induces partial population transfer from the dark  state to the symmetric superposition |*T*_0_⟩ = (|↑↓⟩ + |↓↑⟩)/√2. Hence, the EPR signal shows emission lines for 2–1 and 3–1 transitions and absorptions for 2–4 and 3–4. Conversely, an initial ρ_*p*_ state (polarized along the chiral axis) is strongly anisotropic. For a generic orientation of the molecule with respect to the external field (which defines the quantization axis) such a state has sizable components on states |↑↑⟩ and |↓↓⟩. If these components are larger than that on |*T*_0_⟩, we get emission lines for 4–2 and 4–3 transitions and absorption for 1–2 and 1–3. This situation (opposite to that of the singlet) dominates on the spherical average (see the Supporting Information for details). Then, the order of maxima and minima is determined by the sign of the spin–spin interaction (fixed by considering a dipolar coupling).

**Figure 4:**
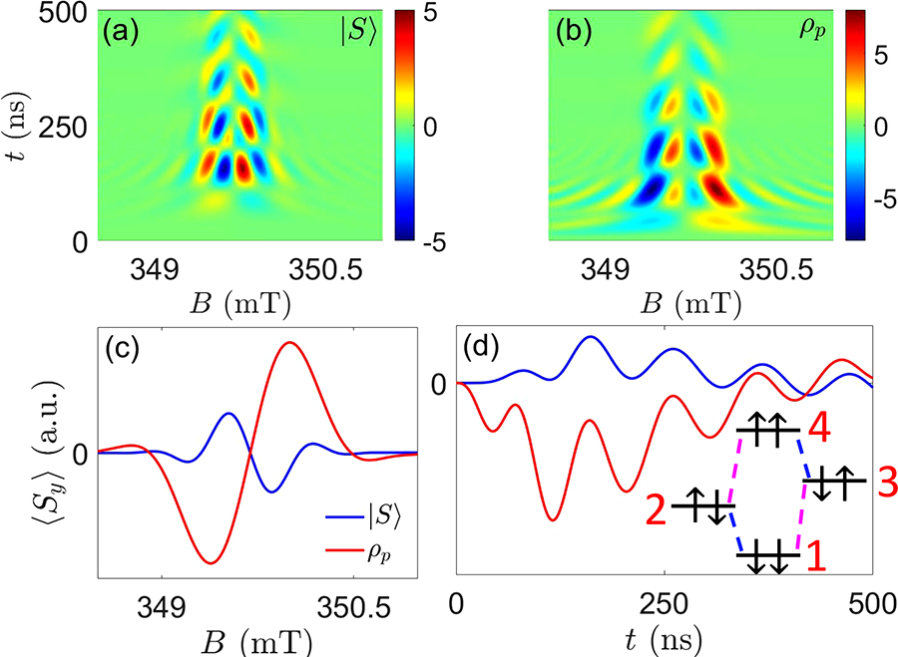
TR-EPR on a randomly oriented ensemble of D-χ-A molecules. Parameters: Δ*g* = 0.002, *r*_DA_ = 25 Å, *hν* = 9.8 GHz. (a) and (b) Two-dimensional maps of ⟨*S*_*y*_(*t*, *B*)⟩ for an initial singlet or polarized state, respectively. (c) Field dependence of the absorption TR-EPR spectrum, integrated in the time-window corresponding to the first maxima-minima in the maps of panels (a) and (b), for the states , ρ_*p*_ (with either *p* = −1 or *p* = 1, leading to the same result in solution). (d) Time dependence around *B* ≈ 349.5 mT, highlighting the opposite behavior at short times for polarized and unpolarized states. Simulations include relaxation, dephasing, and recombination of the radical pair, with *T*_1_ = 2 μs, *T*_2_ = 0.5 μs, *T*_*R*_ = 10 μs (in the singlet–triplet RP basis, see the Supporting Information), and Gaussian broadening with fwhm = 0.15 mT.^36^ Inset: schematic energy-level diagram, with states practically corresponding to eigenstates of *S*_*zi*_. Allowed EPR transitions of D (A) are indicated by blue (purple) dashed lines.

We finally note that, because the spectrum in panel b is the same for any choice of *p*, this measurement does not probe the acceptor polarization but distinguishes a factorized state ρ_*p*_ from a singlet. As shown in the Supporting Information, unpolarized states |ψ_*U*_⟩ give different patterns, intermediate between  and ρ_*p*_, thus requiring, in general, a preliminary characterization of the system Hamiltonian to clearly reveal CISS.

*Identification of a Suitable D-χ-A-Q*. The identification of a suitable D-χ-A-Q requires the optimization of many factors, including ET efficiency and stability of the helicoidal structure in solution, but appears within reach. Considering the individual building blocks, D–A dyads providing long-lived radical pairs are available in the literature.^72,73^ The most commonly used acceptors are C_60_ or derivatives of naphthalenediimide, whereas pyrene, oligophenylene-vinylene, and tetrathiafulvalene are suitable donors. Linkers employed in these dyads are usually short and strongly conjugated and thus significantly more conductive than foldamers based on polypeptides commonly used to detect CISS in transport measurements.^1,2^ Interestingly, the strong dipole moment of α helices has been shown to enhance intramolecular ET. Long distance ET, up to 5 nm, has been instead observed in D-χ-A units based on helically folded oligoamide of 8-amino-2-quinolinecarboxylic acid.^74^ However, both forms of handedness are present and interconversion is relatively fast in solution. Nevertheless, racemization can be hampered in more rigid helicoidal scaffolds such as helicene. Condensation of a D–A unit with a radical as a qubit has been recently achieved.^64,75^ In our case, where pulses separately addressing the photogenerated radical and the permanent qubit are required, transition metal-based qubits with long coherence^42,44−58^ are preferred because of their *g* value differing from 2, as discussed above.

In conclusion, we have proposed simple magnetic resonance experiments exploiting a qubit as a probe of the acceptor polarization in electron-transfer processes through a chiral bridge. These experiments will ultimately unravel the nature of chiral-induced spin selectivity at the single-molecule level. We finally note that, by applying the proposed sequence for polarization transfer, the CISS effect could be exploited for initialization and read-out of the qubit state, an alternative to optical initialization recently achieved in a Cr^4+^*S* = 1 complex.^76^ This is a crucial step toward the physical implementation of quantum computers. A CISS-based approach would be more costly in terms of chemical engineering, but it could reveal tremendous potential. Indeed, the photoexcitation step could be replaced by electrically induced CISS-ET and combined with electric read-out.

## Supporting Information Available

Overview of state-of-the-art experiments on CISS, details on the simulation of time-resolved EPR spectra on D-χ-A-Q and D-χ-A systems, additional simulations with different parameter set and including the effect of isotropic exchange and of experimental imperfections, and details on the calculation of NMR spectra (PDF)
