# Supplementary material for: Assessing the Nature of Chiral-Induced Spin Selectivity by Magnetic Resonance
Source: J Phys Chem Lett. 2021 Jul 6;12(27):6341–7. doi: 10.1021/acs.jpclett.1c01447 (PMC8397348; doi:10.1021/acs.jpclett.1c01447)
Supplement: Supplementary file 1 — jz1c01447_si_001.pdf [file jz1c01447_si_001.pdf]

Supporting Information for: Assessing the Nature of Chiral-Induced Spin Selectivity by Magnetic Resonance

A. Chiesa,[1,2] M. Chizzini,[1] E. Garlatti,[1,2] E. Salvadori,[3] F. Tacchino,[4] P. Santini,[1,2] I. Tavernelli,[4] R. Bittl,[5] M. Chiesa,[3] R. Sessoli,[6] and S. Carretta[1,2]

[1]*Dipartimento di Scienze Matematiche, Fisiche e Informatiche, Università di Parma, I-43124 Parma, Italy*

[2]*UdR Parma, INSTM, I-43124 Parma, Italy*

[3]*Dipartimento di Chimica & NIS Centre, Università di Torino, Via P. Giuria 7, I-10125 Torino, Italy*

[4]*IBM Quantum, IBM Research – Zurich, Säumerstrasse 4, 8803 Rüschlikon, Switzerland*

[5]*Freie Universität Berlin, Fachbereich Physik, Berlin Joint EPR Lab, Arnimallee 14, D-14195 Berlin, Germany*

[6]*Dipartimento di Chimica "Ugo Schiff" & INSTM, Università Degli Studi di Firenze, I-50019 Sesto Fiorentino*

I. STATE-OF-THE-ART EXPERIMENTS ON CISS

We provide below a brief overview of some important experiments on CISS. Although most measurements have probed the current polarization in a transport setup, evidences of CISS have been found also without net transport yielding a stationary current [S1].
We focus here on this class of experiments, in order to concentrate on qualitative features emerging directly from chiral molecules. Although some theoretical works have proposed a crucial role played by initial and final unbound states [S2, S3], typical of transport measurements, a bunch of experiments studying $\chi-$SAMs on a conducting substrate indicate that polarization occurs also in very different contexts, in which chiral molecules are in a *static* configuration, i.e. not crossed by a stationary current. These experiments include magnetization switching by enantiomer deposition [S4], selection of enantiomer adsorption by the polarization of a FM substrate [S5], or Kelvin-probe measurements on FM electrodes covered by a $\chi-$SAM, indicating that a strong interaction exists between chiral molecules and a FM substrate and suggesting that penetration of electron spin wave-function in $\chi-$SAM is enantiospecific [S6, S7]. Most relevant, a Hall potential was detected in a two-dimensional electron gas coated with a $\chi-$SAM subject to an electric field [S8, S9]. The latter induced a charge polarization and a consequent spin polarization [S10], yielding the Hall effect on the conducting electrons. Within the same apparatus, microwave absorption was measured, with a sign depending on the direction of the applied electric pulse [S9]. Last but not least, electron transfer was found to be spin selective in experiments performed on Photosystem-I [S11].
All of these experiments indicate CISS as a property of individual chiral molecules, not arising accidentally from specific initial conditions. In particular, they seem to exclude a role of the leads or of the peculiar initial state of transport setups in the final spin polarization.

II. TIME-RESOLVED EPR SPECTRA

A. Theory

Time-resolved EPR spectra are computed according to the formalism developed, e.g. in Refs. [S12–S15]. The system is described by spin Hamiltonian (1) of the main text. The TR-EPR spectrum corresponds to the calculation of $\langle S_y(B,t)\rangle$ in a reference frame rotating

at the frequency of the oscillating magnetic field $\mathbf{B}_1(t) = B_1(\cos\omega t\,\hat{\mathbf{x}} + \sin\omega t\,\hat{\mathbf{y}})$ about the static field $\mathbf{B}$. In such a reference frame and by neglecting non-secular terms (as usually done in the high-field limit) the explicit time-dependence is eliminated from the system Hamiltonian, which reduces to

$$\begin{aligned} H_{\rm rot}(\Omega) = & \sum_i (\mu_B B g_i^{zz}(\Omega) - \hbar\omega) S_i^z \\ & + \sum_{i,j} J_{ij}^{zz}(\Omega)(2S_i^z S_j^z - S_i^x S_j^x - S_i^y S_j^y) \\ & + \bar{g}\mu_B B_1 \sum_i S_i^x . \end{aligned} \tag{S1}$$

Here we have also assumed an average isotropic coupling to the oscillating field, with $\bar{g}$, as reasonable for $B_1 \ll B$. [In our simulations we have assumed $B_1 = 0.03$ mT].

In general, the parameters g_i^{zz} and J_{ij}^{zz} of $H^{\rm rot}$ depend on the orientation of the molecule with respect to the static field (indicated by the set of Euler angles Ω, see Ref. [S13] for the full transformation from the local to the laboratory frame). In the case of randomly-oriented solutions, spherical average over Ω must be included in the calculation.

Besides evolution induced by Hamiltonian (S1), we have included in our simulations incoherent terms modeling relaxation, dephasing and recombination processes. The resulting stochastic Liouville equation is given by

$$\frac{\partial \rho_r(\Omega,t)}{\partial t} = -i\left[\frac{1}{\hbar}\tilde{H}(\Omega) + i\tilde{R} + i\tilde{K}\right]\rho_r(\Omega,t), \tag{S2}$$

where $\rho_r(\Omega,t)$ denotes the system density matrix in the rotating frame, $\tilde{H}(\Omega)$, $\tilde{R}$ and $\tilde{K}$ are super-operators associated to Hamiltonian (S1) and to phenomenological relaxation/decoherence and recombination mechanisms, respectively. Note that in our simulations both $\tilde{H}$ and the initial state $\rho_r(\Omega,0)$ depend on Ω. In particular, initial states subject to CISS are polarized along the molecular chiral axis (not corresponding to z in a randomly-oriented solution). Examples for specific orientations are discussed below.

We are here neglecting various rotational processes (slow-motional limit). Following Ref. [S12], when describing the RP, we have assumed the same relaxation rate ($1/T_1$) for each diagonal element of ρ_r and the same dephasing rate ($1/T_2$) for each off-diagonal element, i.e. $\tilde{R}_{iijj} = 1/T_1$ and $\tilde{R}_{ijij} = -1/T_2$ in the singlet-triplet basis. Conversely, simulations on the D-χ-A-Q system in Q-band are performed by treating relaxation and dephasing processes independently for the three spins, as in Ref. [S16]. By imposing the stationary condition

$\sum_j R_{jjii} = 0$ diagonal elements of $\tilde{R}$ are determined. Finally, the recombination superoperator is given by $\tilde{K}_{ijkl} = -1/T_R(\delta_{ik}\delta_{jl} + \delta_{ij}\delta_{ik})$, with $1/T_R$ the charge recombination rate. Equation (S2) can be formally integrated, leading to

$$\rho_r(\Omega, t) = \exp\left\{\left[-\frac{i}{\hbar}\tilde{H}(\Omega) + \tilde{R} + \tilde{K}\right] t\right\} \rho_r(\Omega, 0) \tag{S3}$$

from which $\langle S_y(B,t)\rangle = \mathrm{Tr}[\rho_r(\Omega,t)S_y]$ can be computed.

Hyperfine coupling of the probed electronic spins with surrounding nuclear spins is expected to broaden oscillations in the time dependence of $\langle S_y(B,t)\rangle$. In the field-dependent spectrum, both the coupling to surrounding nuclear spins and inhomogenous broadening of the system parameters are included by convoluting spectra with a Gaussian line shape function [S14, S15]. A more detailed treatment of the hyperfine coupling would require knowledge of the specific system under investigation and is therefore beyond the scope of the present work.

B. TR-EPR on D-χ-A-Q system

We show in Fig. S1 the TR-EPR spectra for the initial states reported in the main text, as a function of both B and t, along with the level diagram indicating the probed excitations. Note that in these simulations the effect of dephasing, relaxation and charge recombination is overcome by the gaussian broadening of the peaks (FWHM 2.35 mT), a safe assumption for both the molecular qubit and the RP, even at room temperature. Other effects Interaction with surrounding nuclei could, in addition, broaden time-dependent oscillations. These features depend on the specific examined system and hence are not included in the calculation, but do not alter our conclusions.

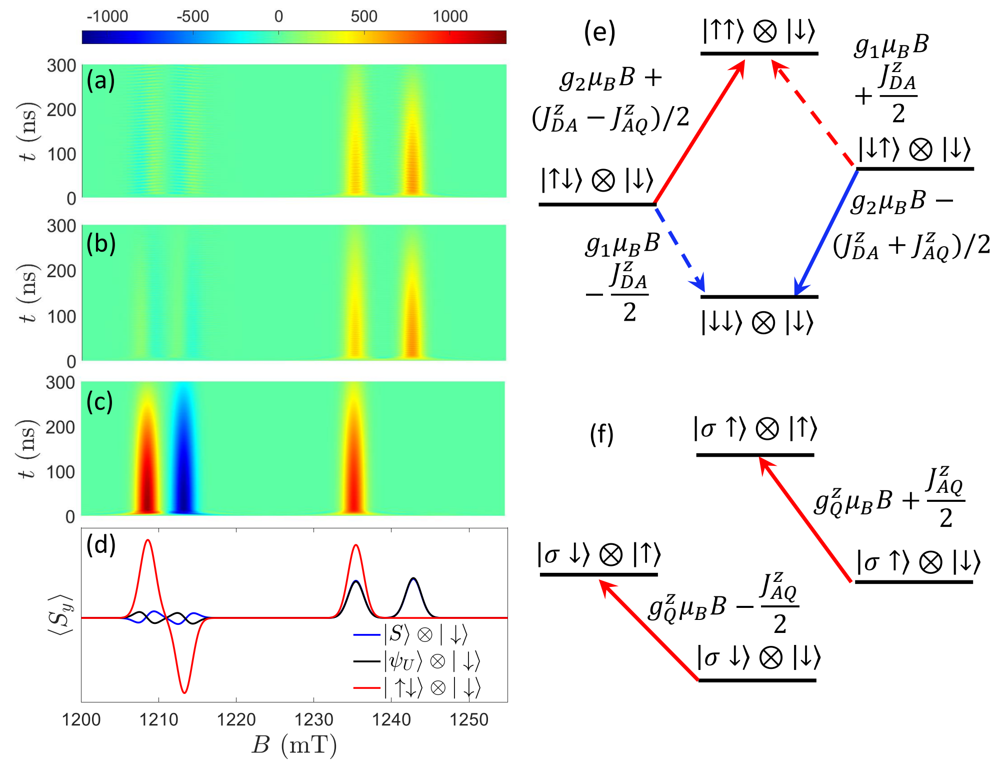

FIG. S1. Time-resolved EPR on D-χ-A-Q. (a-c) $\langle S_y(B,t)\rangle$ with an initial singlet state of the radical pair (a), a generic un-polarized state $|\psi_U(\pi,\lambda,\phi)\rangle$ (b) and a polarized $|\uparrow\downarrow\rangle$ state (c). (d) $B-$dependence of the TR-EPR signal, integrated between 100 and 300 ns. Inhomogeneous broadening effects included with gaussian line broadening 2.35 mT. (e) Schematic representation of the energy level of the system, focusing on excitations of the D-χ-A radical-pair, with arrows indicating energy gaps corresponding to absorption (red) and emission (blue) peaks shown in panel (a) at low field. Continuous (dashed) arrows correspond to excitations of acceptor (donor), characterized by isotropic $g_{1,2} = g_e \mp \Delta g/2$, with $\Delta g = 0.002$. The static field is parallel to the chiral axis (labeled by z) and in the examined range of parameters $\Delta g \mu_B B >> J^{x,y}$ and hence eigenstates practically correspond to the factorized states indicated on top of the levels, with energy gaps to first order also reported. (f) As in (e), focusing on qubit transitions. These are independent of the state of D, indicated generically as σ, but depend on the the state of A via the coupling $\mathbf{J}_{AQ}$.

1. *Spectra for different initial states*

In Fig. S2 we report TR-EPR spectra on different initial states $|\psi_U(\theta, \phi, \lambda)\rangle$ and different amount of polarization for the state ρ_p. As stated in the main text, different choices of the rotation angles (θ, ϕ, λ) modify the spectrum of the radical pair, but yield practically the same response of the qubit probe (at high field). We also point out that the response of the qubit is substantially the same for all un-polarized states (compare $p = 0$ case in panel b with all results of panel a).

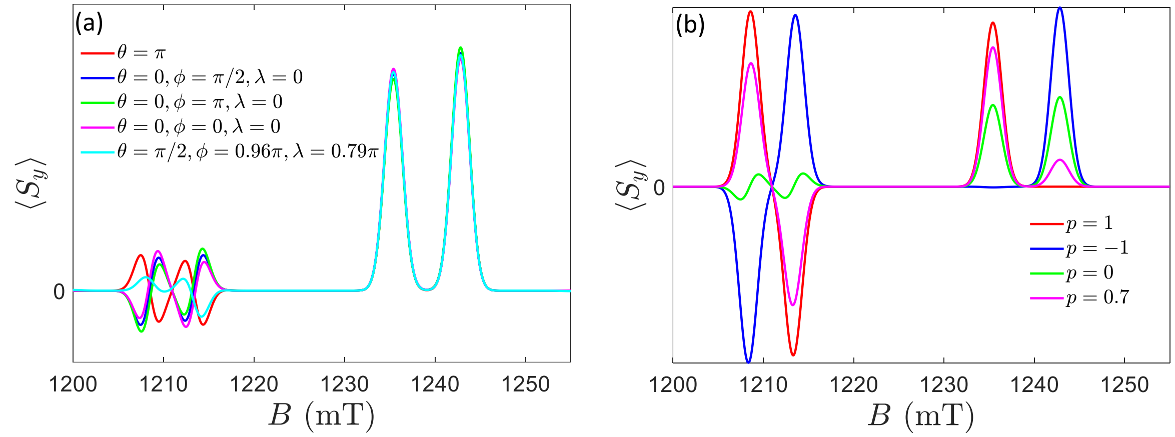

FIG. S2. TR-EPR spectra of D-χ-A-Q for different initial states $\psi_U(\theta, \lambda, \phi)$, including $\theta = 0$ state analysed in Ref. [S3] (a) and for different polarization of the polarized state ρ_p (b).

2. *Effect of A-Q exchange interaction*

We examine here the effect of including an additional isotropic exchange contribution to the acceptor-qubit (A-Q) interaction, assumed to be purely dipolar in the simulations reported in the main text. The simulations reported in Fig. S3 below demonstrate that the specific form of the spin-spin interaction does not influence our conclusions, provided that its transverse component is significantly smaller than $|g_A - g_Q|\mu_B B$. This condition is required by our quantum sensing scheme to avoid entanglement between A and Q, i.e. to use the qubit as a probe which must not significantly perturb the D-χ-A unit. In particular, we consider an additional ferromagnetic isotropic exchange $J_{iso}\mathbf{S}_A \cdot \mathbf{S}_Q$, as expected from a singlet precursor [S17]. For distances in the nm range, we expect J_{iso} to be of similar

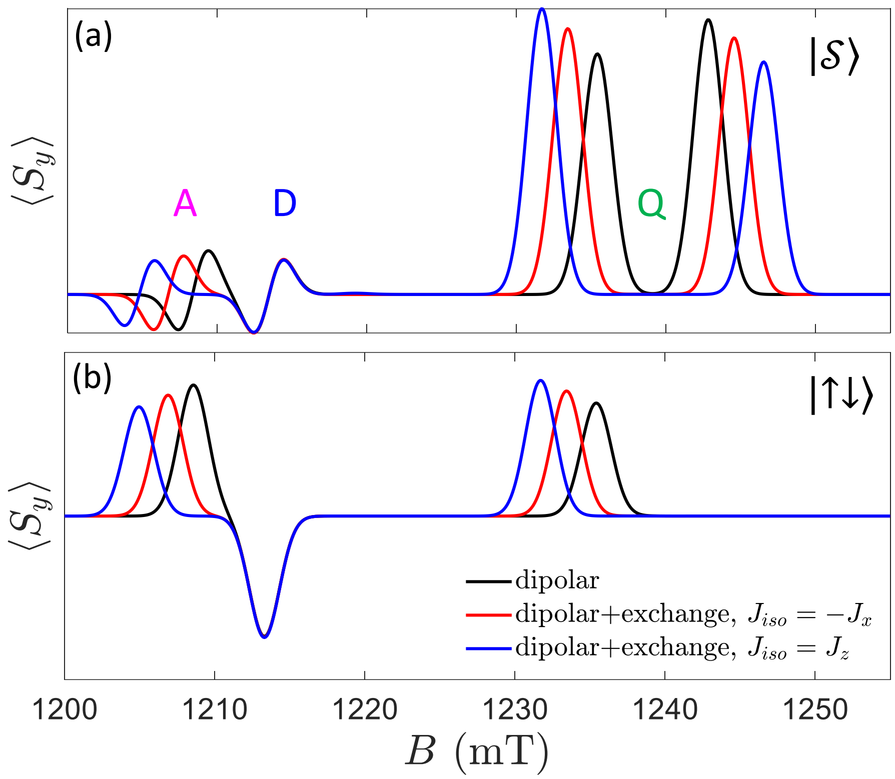

FIG. S3. TR-EPR spectra of D-χ-A-Q (integrated between 100 and 300 ns) for different forms of the spin-spin interaction, initialized in a singlet (a) or in a fully polarized state (b). In these examples, we have chosen a ferromagnetic J_{iso} equal to the transverse ($-J_x$) or longitudinal ($J_z = -2J_x$) component of the dipolar coupling.

magnitude to the dipolar contribution. In Fig. S3, we assume for instance J_{iso} equal to the transverse or longitudinal component of the dipolar coupling. In both cases, starting from the singlet state [Fig. S3-(a)], the splitting of the qubit signal (right part of the spectrum, labeled by Q) is increased by adding the isotropic contribution. Indeed, this splitting depends on the component of the interaction parallel to the external field (z), which is enlarged by a ferromagnetic exchange ($J_{iso} < 0$). Therefore, this additional term does not alter the feasibility of the proposed experiment. Note, in turn, that the acceptor signal (left part of the spectrum, labeled by A) is also slightly moved to the left by increasing the longitudinal component of the spin-spin coupling. The situation is perfectly analogous for a polarized state [panel (b)], but with one of the two qubit lines removed, as discussed in the text.

3. *Effect of experimental imperfections*

We investigate the experimental feasibility of the proposed experiments by analysing the effect of worsening some of the experimental parameters. We consider, in particular inhomogeneous broadening of the system parameters, decoherence related to relaxation and dephasing, charge recombination and efficiency of CISS. Inhomogenous broadening is accounted for by convoluting the signal with a gaussian line-shape. In the text we assumed a rather large FWHM of 2.35 mT, which could be safely reduced to 1 mT [S18], thus producing the blue spectrum of Fig. S4-(a).

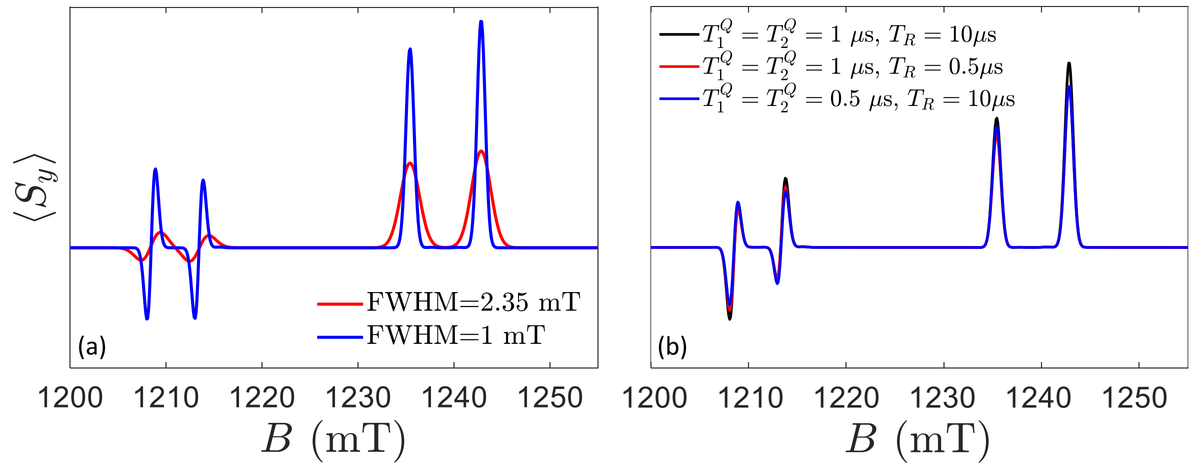

FIG. S4. TR-EPR spectra of D-χ-A-Q (integrated between 100 and 300 ns) for different gaussian broadenings of the peaks (a) and for different values of the relaxation and dephasing parameters of the qubit (b), assuming a FWHM of 1 mT and starting from a singlet state. Analogous effects are obtained starting from a polarized state.

Relaxation and dephasing on the qubit (parametrized by T_1^Q and T_2^Q, respectively) can induce a further broadening of the peaks at high field. Although values of $T_1^Q, T_2^Q \sim$ few μs are reasonable for VO-based molecular qubits even at room temperature [S19], reducing both of them to hundred of ns does not significantly influence the spectrum [blue line in Fig. S4-(b)]. Analogous considerations hold for the charge recombination time T_R, which can be downgraded from 10 to 0.5 μs without affecting our conclusions [red line in Fig. S4-(b)]. Note that here we have assumed lines narrower than in the main text, in order to highlight the effect of other incoherent parameters. With the safe choice of FWHM=2.35 mT adopted

in the paper, all these effects would be overcome by inhomogeneous broadening.

CISS efficiency translates into a value of $|p| < 1$ in the polarized state $\rho_p = \frac{1+p}{2}|\uparrow\downarrow\rangle\langle\uparrow\downarrow| + \frac{1-p}{2}|\downarrow\uparrow\rangle\langle\downarrow\uparrow|$. It affects the relative intensities of the two peaks of the qubit (corresponding to the two polarizations of the acceptor), as shown in Fig. S5. In practice, the relative intensity of the peaks is a direct measurement of the efficiency of CISS. We finally mention that

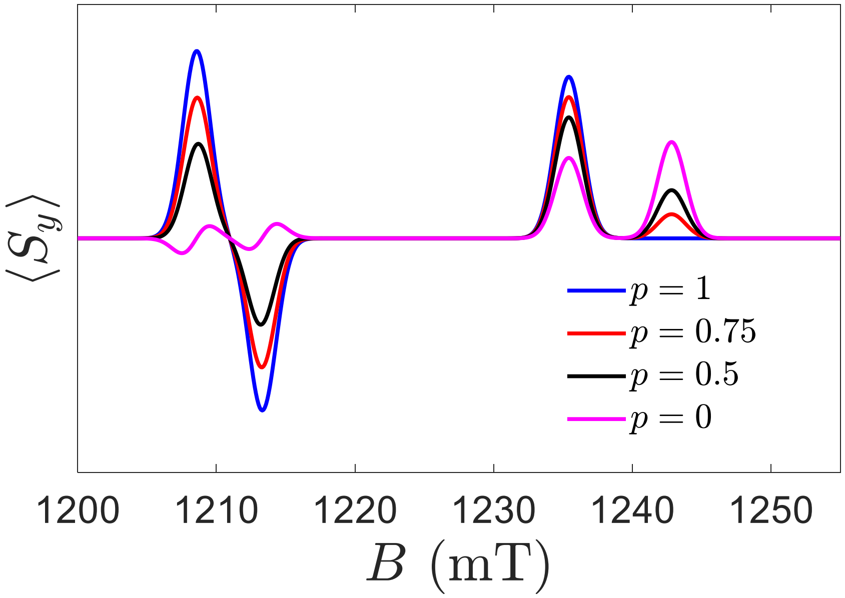

FIG. S5. TR-EPR spectra of D-χ-A-Q (integrated between 100 and 300 ns) for different CISS efficiency, identified by the polarization p of the initial state $\rho_p = \frac{1+p}{2}|\uparrow\downarrow\rangle\langle\uparrow\downarrow| + \frac{1-p}{2}|\downarrow\uparrow\rangle\langle\downarrow\uparrow|$.

an important time-scale is that related to the charge-transfer process. In the here-studied systems, we assume a fast single-step ET (much faster than T_1 and T_2) from D to A with D and A having a well-defined static geometry. This allows us to neglect the dynamics of the bridge and to study the spin state of the system after ET.

C. TR-EPR of randomly oriented D-χ-A

1. *Order of the peaks*

In order to clarify the different pattern of maxima and minima observed Fig. 4 of the main text, we provide here an analysis of the spectra along specific directions. We consider an initial state fully-polarized along the chiral axis, i.e. represented by $|\uparrow\downarrow\rangle$ in the *molecular* frame. Its form in the laboratory reference frame (with the z axis parallel to the external field) is obtained by a proper rotation. In particular, for the symmetry directions we find

$$|\psi_0^{\pm x}\rangle = e^{\mp i S_y \pi/2}|\uparrow\downarrow\rangle = \frac{|\mathcal{S}\rangle}{\sqrt{2}} \pm \frac{|\uparrow\uparrow\rangle - |\downarrow\downarrow\rangle}{2} \tag{S4}$$

$$|\psi_0^{\pm y}\rangle = e^{\mp i S_x \pi/2}|\uparrow\downarrow\rangle = \frac{|\mathcal{S}\rangle}{\sqrt{2}} \pm i\frac{|\uparrow\uparrow\rangle + |\downarrow\downarrow\rangle}{2} \tag{S5}$$

$$|\psi_0^{z}\rangle = |\uparrow\downarrow\rangle, \quad |\psi_0^{-z}\rangle = |\downarrow\uparrow\rangle \tag{S6}$$

Starting from each of these states, we compute the fields corresponding to absorptions (B_a) and emissions (B_e). We consider $g_1 < g_2$ and $\Delta g \mu_B B < J$, as in the scheme of the inset of Fig. 4(d) in the main text. Along z, we find

$$B_a = \frac{\hbar\omega}{g_{1,2}\mu_B B} - \frac{J_z}{2g_{1,2}\mu_B B} \tag{S7}$$

$$B_e = \frac{\hbar\omega}{g_{1,2}\mu_B B} + \frac{J_z}{2g_{1,2}\mu_B B}, \tag{S8}$$

corresponding to transitions $2 \to 4$, $3 \to 4$ for absorption and $2 \to 1$, $3 \to 1$ for emission in the scheme of Fig. 4(d). This means that, for each of the two spins, $B_a > B_e$ because $J_z < 0$.

Conversely, along x or y we get

$$B_a = \frac{\hbar\omega}{g_{1,2}\mu_B B} + \frac{J_{x,y}}{2g_{1,2}\mu_B B} \tag{S9}$$

$$B_e = \frac{\hbar\omega}{g_{1,2}\mu_B B} - \frac{J_{x,y}}{2g_{1,2}\mu_B B}, \tag{S10}$$

corresponding to transitions $1 \to 2$, $1 \to 3$ for absorption and $4 \to 2$, $4 \to 3$ for emission. Again, for each spin we find $B_a > B_e$ because absorption/emission are exchanged but also $J_{x,y} > 0$. Hence, also in the spherical average we have $B_a > B_e$ for this initial state. We finally point out that, due to the peculiar out-of-equilibrium state considered, the angular average must be computed on the whole sphere and not on a single hemisphere.

2. *Spectra for different initial states*

Fig. S6 below shows TR-EPR spectra simulated for different initial states, corresponding to different sets of (θ, λ, ϕ) in $|\psi_U\rangle$. All simulations include relaxation, dephasing and recombination of the radical pair, with $T_1 = 2$ μs, $T_2 = 0.5$ μs, $T_R = 10$ μs, and gaussian broadening with FWHM = 0.15 mT. Panel (a) correspond to state $(|\uparrow\uparrow\rangle + |\downarrow\downarrow\rangle)/\sqrt{2}$, panel (b) is $(|\uparrow\uparrow\rangle - |\downarrow\downarrow\rangle + |\uparrow\downarrow\rangle + |\downarrow\uparrow\rangle)/2$, panel (c) is the symmetric superposition $|T_0\rangle = (|\uparrow\downarrow\rangle \pm |\downarrow\uparrow\rangle)/\sqrt{2}$, while state (d) is the one considered in Ref. [S3] and similar to the singlet.

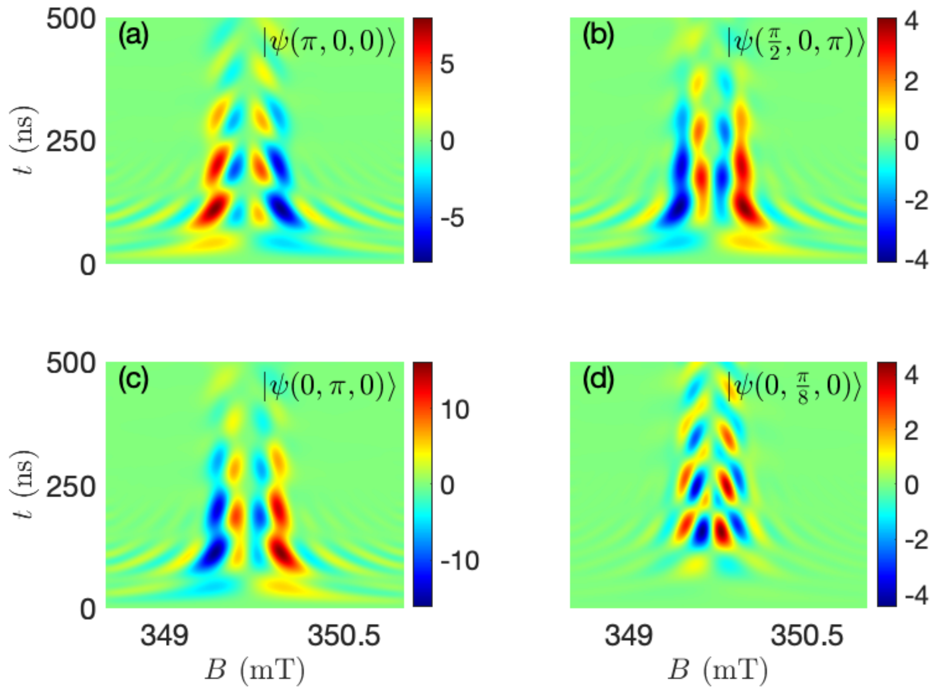

FIG. S6. TR-EPR on D-χ-A in solution for different initial states $|\psi_U(\theta, \lambda, \phi)\rangle$. Parameters: $\Delta g = 0.002$, $r_{DA} = 25$ Å, $h\nu = 9.8$ GHz.

3. *Spectra for different parameter sets*

We have then studied the effect of different choices of the system parameters on the EPR spectrum. This study could be important to guide synthetic efforts, in order to design proper experimental condition to highlight CISS. We remark that general considerations reported in the main text on the order of absorption/emission peaks remain valid even for these different parameter sets (see Figs. S7,S8,S9). In general, especially for anisotropic g

values, the spectrum is more complex and understanding the dependence on the initial state requires a precise characterization of the system Hamiltonian.

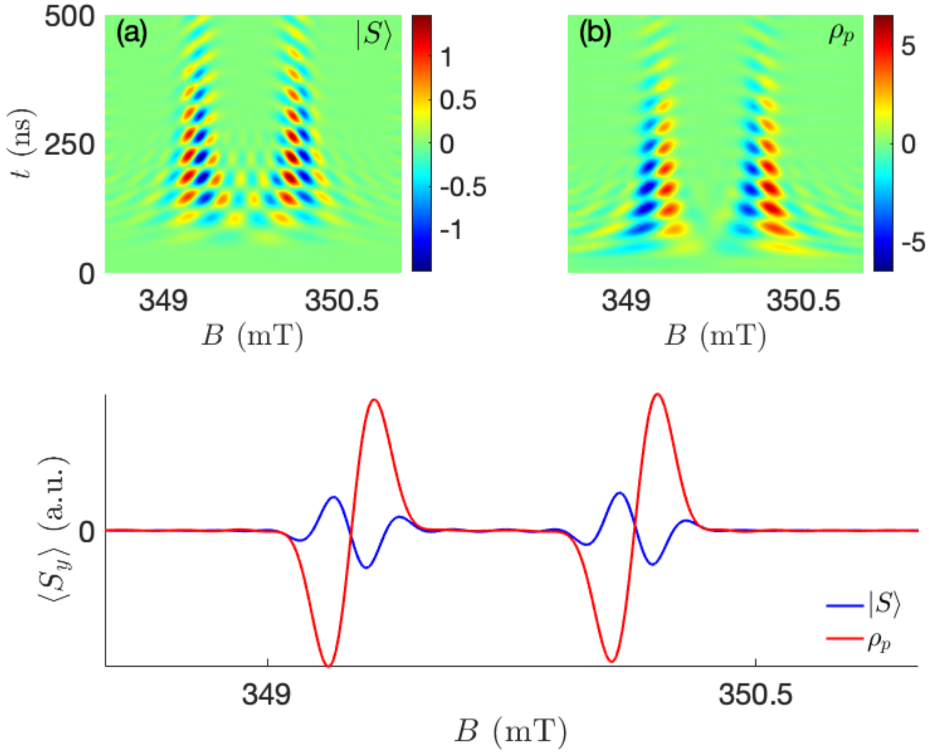

FIG. S7. TR-EPR on a randomly oriented ensemble of D-χ-A molecules. Parameters: $\Delta g = 0.005$, $r_{DA} \sim 29$ Å, $h\nu = 9.8$ GHz. (a,b) Two-dimensional maps of $\langle S_y(t, B) \rangle$ for an initial singlet or polarized state, respectively. (c) Field dependence, integrated from 200 to 500 ns.

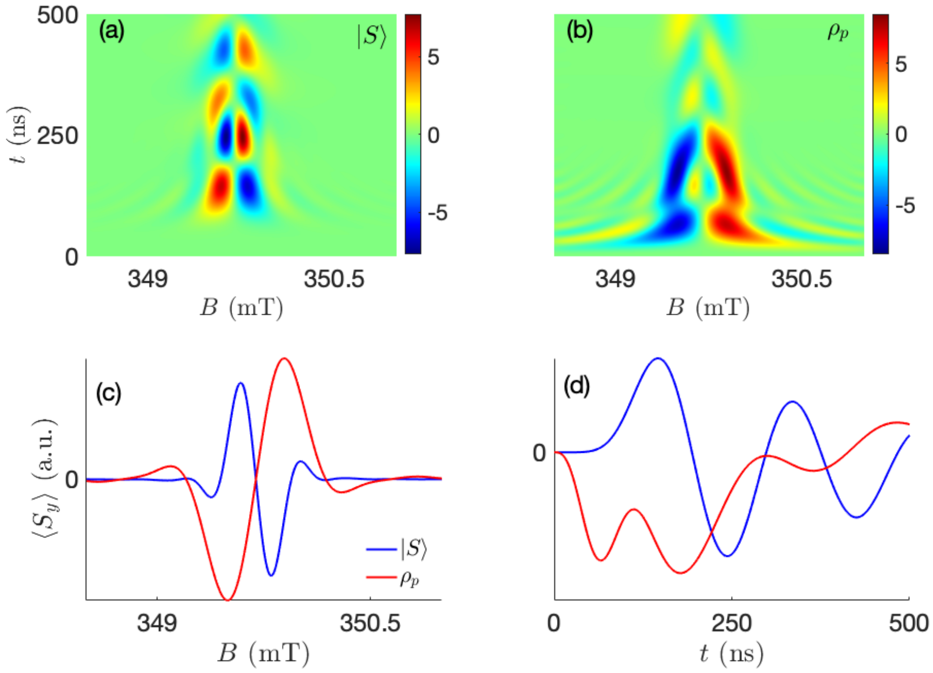

FIG. S8. TR-EPR on a randomly oriented ensemble of D-χ-A molecules. Parameters: $\Delta g = 0.001$, $r_{DA} \sim 23$ Å, $h\nu = 9.8$ GHz. (a,b) Two-dimensional maps of $\langle S_y(t, B) \rangle$ for an initial singlet or polarized state, respectively. (c) Field dependence of the absorption TR-EPR spectrum, integrated in the time-window corresponding to the first maxima-minima in the maps of panels (a,b). (d) Time dependence around $B \sim 349.5$ mT, highlighting the opposite behavior at short times for polarized and un-polarized states.

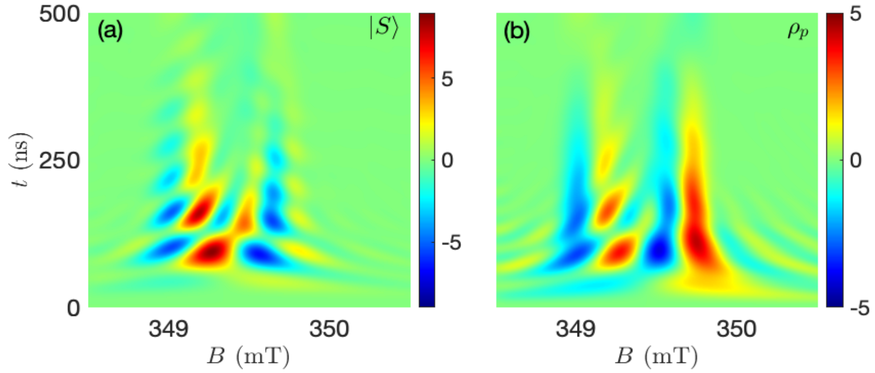

FIG. S9. TR-EPR on a randomly oriented ensemble of D-χ-A molecules. Parameters: anisotropic $\mathbf{g}_i$ with principal values $\mathbf{g}_1 = [2.00285, 2.00285, 2.0022]$, $\mathbf{g}_2 = [2.0061, 2.00485, 2.0027]$, and relative orientation as in Ref. [S20], $r_{DA} \sim 29$ Å, $h\nu = 9.8$ GHz.

D. Effect of experimental imperfections

We analyze below the impact of worsening some parameters on the TR-EPR spectra of a randomly oriented solution of D-χ-A molecules, as done for the oriented D-χ-A-Q systems. In particular, in Fig. S10 we show the effect of increasing the gaussian line-width from 0.15 to 0.3 mT, while in Fig. S11 we consider halved T_1 and T_2 and T_R reduced of an order of magnitude. In general, as far as all these parameters are above a few hundreds of ns, their precise value does not qualitatively modify our conclusions.

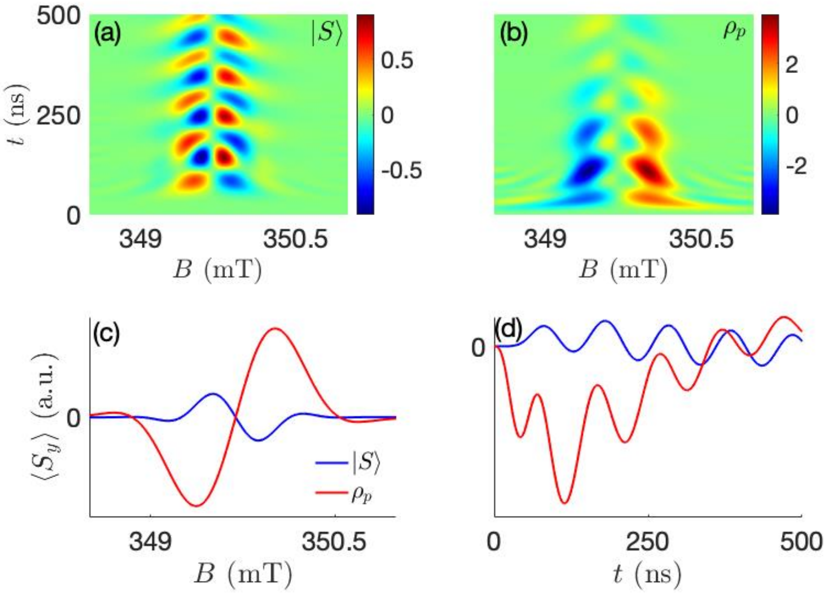

FIG. S10. TR-EPR on a randomly oriented ensemble of D-χ-A molecules, with the same parameters of Fig. 4 in the main text, except an increased gaussian line-width of FWHM = 0.3 mT.

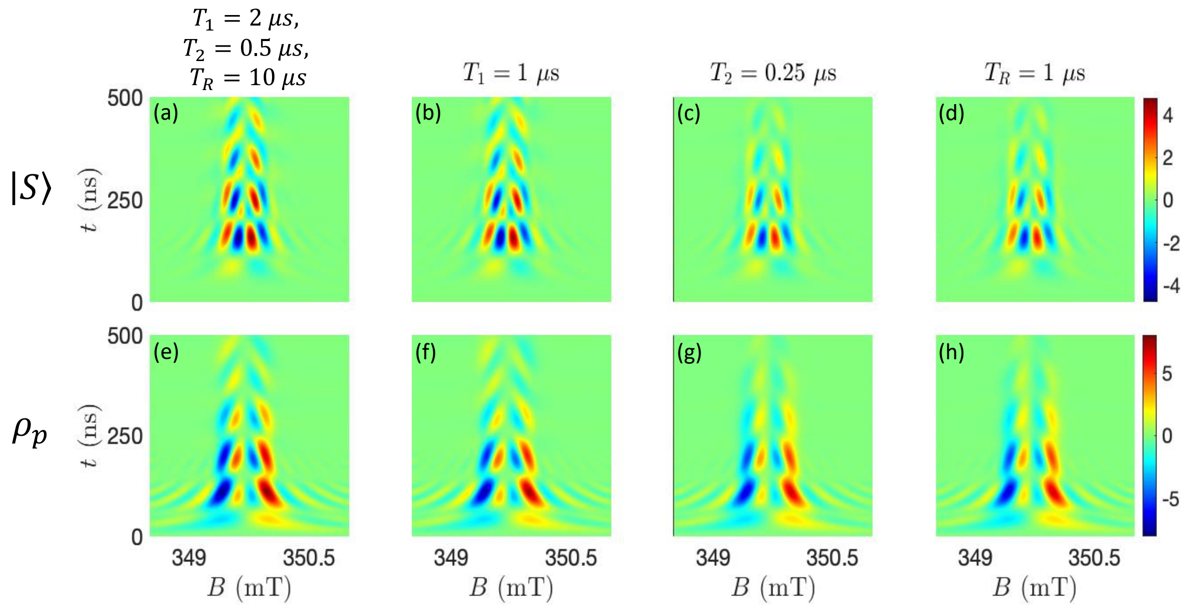

FIG. S11. TR-EPR on a randomly oriented ensemble of D-χ-A molecules for different values of the decoherence parameters. Panels (a-d) refer to a singlet initial state, panels (e-h) to a polarized initital state ρ_p. In the first column (a,e) we report the simulated time and field dependence of the spectrum, with paarmeters used in the main text. In the next columns we compare the effect of lowering relaxation, dephasing and recombination times, separately: in panels (b,f) T_1 is halved, in panels (c,g) T_2 is halved and in panels (d,h) T_R is reduced by a fcactor 10.

III. NMR SPECTRUM

The NMR spectrum is proportional to the dynamic susceptibility of the system [S21]:

$$\chi''_{\alpha\beta}(\omega) \propto \sum_{\xi\xi'} \frac{\langle\xi|M_\alpha|\xi'\rangle\langle\xi'|M_\beta|\xi\rangle}{(E_{\xi'} - E_\xi - \hbar\omega)^2 + \Lambda^2}\Lambda\ (n_\xi - n_{\xi'}) \tag{S11}$$

with $|\xi\rangle$ the eigenstates of the system, $M_\alpha = \sum_i g_i S_i^\alpha$ the magnetic moment perpendicular to the static field and ω in the radio-frequency range. Here we have assumed a Lorentzian lineshape of width Λ.

$\chi''(\omega)$ peaks at the frequency corresponding to excitation of the probed nucleus (corresponding to its Larmor frequency shifted and/or splitted by interactions with neighboring spins). In the simulations reported in the main text we have assumed that the time required for measuring NMR spectra (few μs) is shorter than the recombination and relaxation times of the radical pair ($\sim 10\ \mu$s), but longer than T_2 ($\sim 0.5\ \mu$s), thus making coherences irrelevant.

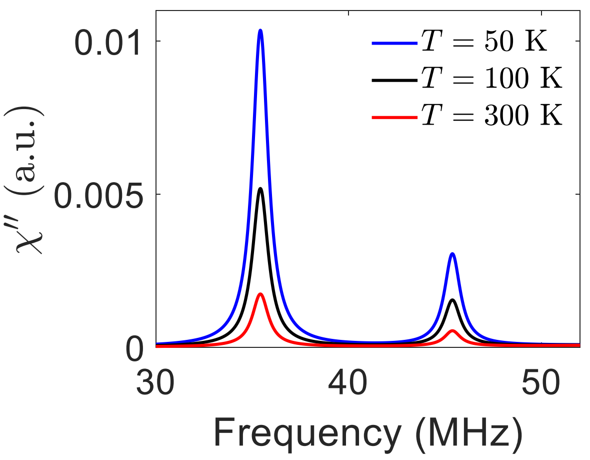

FIG. S12. **NMR spectrum on D-χ-A-I at different temperatures**, for a partially polarized DA state ($p = 0.7$).

A. Effect of temperature

We report below the effect of temperature on the NMR spectrum of a nuclear spin $I = 1/2$ coupled to the acceptor by an isotropic hyperfine interaction $\mathcal{A} = 10$ MHz (see main text). A similar analysis also applies to the EPR case. We assume the state of the system (after ET) is described by the density matrix $\rho = \rho_p \otimes \rho_I$, where ρ_p is the state of the (partially) polarized DA radical pair, $\rho_p = \frac{1+p}{2}|\uparrow\downarrow\rangle\langle\uparrow\downarrow| + \frac{1-p}{2}|\downarrow\uparrow\rangle\langle\downarrow\uparrow|$, while $\rho_I = e^{-\gamma B I_z/k_B T}/\mathrm{Tr}[e^{-\gamma B I_z/k_B T}]$ is the state of the nuclear spin (at thermal equilibrium before ET). Thermal relaxation occurs on a time scale significantly longer than the NMR acquisition time. In this regime, the effect of increasing temperature is an overall attenuation of the signal, which does not alter the relative intensity of the two peaks, representing excitation of the nuclear spin depending on the state of the acceptor spin. Hence, the relative intensity of the two peaks is a measure of the acceptor polarization, independently of T.

[S1] R. Naaman, Y. Paltiel, and D. H. Waldeck, The Journal of Physical Chemistry Letters **11**, 3660 (2020).

[S2] S. Dalum and P. Hedegård, Nano Letters **19**, 5253 (2019).

[S3] T. P. Fay, The Journal of Physical Chemistry Letters **12**, 1407 (2021).

[S4] O. Ben Dor, S. Yochelis, A. Radko, K. Vankayala, E. Capua, A. Capua, S.-H. Yang, L. T. Baczewski, S. S. P. Parkin, R. Naaman, and Y. Paltiel, Nature Communications **8**, 14567 (2017).

[S5] K. Banerjee-Ghosh, O. Ben Dor, F. Tassinari, E. Capua, S. Yochelis, A. Capua, S.-H. Yang, S. S. P. Parkin, S. Sarkar, L. Kronik, L. T. Baczewski, R. Naaman, and Y. Paltiel, Science **360**, 1331 (2018).

[S6] A. Ziv, A. Saha, H. Alpern, N. Sukenik, L. T. Baczewski, S. Yochelis, M. Reches, and Y. Paltiel, Advanced Materials **31**, 1904206 (2019).

[S7] S. Ghosh, S. Mishra, E. Avigad, B. P. Bloom, L. T. Baczewski, S. Yochelis, Y. Paltiel, R. Naaman, and D. H. Waldeck, The Journal of Physical Chemistry Letters **11**, 1550 (2020).

[S8] A. Kumar, E. Capua, M. K. Kesharwani, J. M. L. Martin, E. Sitbon, D. H. Waldeck, and R. Naaman, Proceedings of the National Academy of Sciences **114**, 2474 (2017).

[S9] E. Z. B. Smolinsky, A. Neubauer, A. Kumar, S. Yochelis, E. Capua, R. Carmieli, Y. Paltiel, R. Naaman, and K. Michaeli, The Journal of Physical Chemistry Letters **10**, 1139 (2019).

[S10] R. Naaman, Y. Paltiel, and D. H. Waldeck, Accounts of Chemical Research **53**, 2659 (2020).

[S11] I. Carmeli, K. S. Kumar, O. Heifler, C. Carmeli, and R. Naaman, Angewandte Chemie International Edition **53**, 8953 (2014).

[S12] G. Kothe, S. Weber, R. Bittl, E. Ohmes, M. C. Thurnauer, and J. R. Norris, Chemical Physics Letters **186**, 474 (1991).

[S13] G. Kothe, S. Weber, E. Ohmes, M. C. Thurnauer, and J. R. Norris, The Journal of Physical Chemistry **98**, 2706 (1994).

[S14] S. Weber, T. Biskup, A. Okafuji, A. R. Marino, T. Berthold, G. Link, K. Hitomi, E. D. Getzoff, E. Schleicher, and J. R. Norris, The Journal of Physical Chemistry B **114**, 14745 (2010).

[S15] M. T. Colvin, R. Carmieli, T. Miura, S. Richert, D. M. Gardner, A. L. Smeigh, S. M. Dyar, S. M. Conron, M. A. Ratner, and M. R. Wasielewski, The Journal of Physical Chemistry A **117**, 5314 (2013).

[S16] K. M. Salikhov, S. G. Zech, and D. Stehlik, Molecular Phys. **100**, 1311 (2002).

[S17] M. T. Colvin, R. Carmieli, T. Miura, S. Richert, D. M. Gardner, A. L. Smeigh, S. M. Dyar, S. M. Conron, M. A. Ratner, and M. R. Wasielewski, The Journal of Physical Chemistry A **117**, 5314 (2013).

[S18] L. C. de Camargo, M. Briganti, F. S. Santana, D. Stinghen, R. R. Ribeiro, G. G. Nunes, J. F. Soares, E. Salvadori, M. Chiesa, S. Benci, R. Torre, L. Sorace, F. Totti, and R. Sessoli, Angewandte Chemie International Edition **60**, 2588 (2021).

[S19] M. Atzori, L. Tesi, E. Morra, M. Chiesa, L. Sorace, and R. Sessoli, J. Am. Chem. Soc. **138**, 2154 (2016).

[S20] R. Bittl and G. Kothe, Chemical Physics Letters **177**, 547 (1991).

[S21] J. Jensen and A. R. Mackintosh, *Rare Earth Magnetism* (Clarendon Press, Oxford, United Kingdom, 1991).
